# Differential outcomes of outpatient only versus combined inpatient/outpatient treatment in early intervention for adolescent borderline personality disorder

**DOI:** 10.1007/s00787-023-02222-8

**Published:** 2023-05-11

**Authors:** Marialuisa Cavelti, Nora Seiffert, Stefan Lerch, Julian Koenig, Corinna Reichl, Michael Kaess

**Affiliations:** 1 University Hospital of Child and Adolescent Psychiatry and Psychotherapy, University of Bern, Bolligenstrasse 111, 3000 Bern 60, Switzerland; 2 Department of Child and Adolescent Psychiatry, Centre for Psychosocial Medicine, University of Heidelberg, Heidelberg, Germany; 3 Department of Child and Adolescent Psychiatry, Psychosomatics and Psychotherapy, University of Cologne, Faculty of Medicine and University Hospital Cologne, Cologne, Germany

**Keywords:** Borderline personality disorder, Adolescence, Early intervention, Inpatient, Outpatient, Setting

## Abstract

Clinical guidelines for adults with borderline personality disorder (BPD) recommend outpatient psychotherapy as first-line treatment. Little is known whether this recommendation is also applicable to adolescents. The current study examined the relationship between treatment setting and the outcome of early intervention for adolescents with BPD pathology. One-hundred and seventy-eight adolescents from a specialized outpatient clinic were assessed at baseline, and at 1- and 2-year follow-up. Sixty-three participants who received inpatient treatment during the first year were assigned to the “combined inpatient/outpatient group”, 115 participants to the “outpatient only group”. Generalized linear and mixed models with inverted probability weights to adjust for baseline differences were applied to examine the impact of group on clinical changes over time. Both groups demonstrated a significant decrease in BPD features, depressive symptoms, psychopathological distress, non-suicidal self-injury (NSSI), suicidal thoughts, suicide attempts, and overall illness severity, and a significant increase in quality of life and psychosocial functioning from baseline to follow-up 2. The decrease in NSSI and overall illness severity, and the increase in psychosocial functioning from baseline to follow-up 1 were greater in the outpatient only group, with comparable improvements between groups from follow-up 1 to follow-up 2. Both outpatient treatment and combined outpatient/inpatient treatment resulted in clinical improvements over time, with some indication for faster changes in the outpatient only setting. The findings provide preliminary evidence that the recommendation of outpatient psychotherapy as the first-line treatment for BPD also holds true for adolescents.

**Supplementary Information:**

The online version contains supplementary material available at 10.1007/s00787-023-02222-8.

## Introduction

Borderline personality disorder (BPD) is a severe mental disorder that is characterized by disturbances in identity and interpersonal functioning, emotion regulation deficits, and impulsive and self-harming behaviour [1]. BPD usually emerges during adolescence, peaks in early adulthood, and attenuates over the adult years [2]. It affects around 1.5% adolescents aged 16 years and 3% of young adults aged 22 from the general population, with much higher prevalence rates in clinical samples [3]. The presence of borderline pathology during adolescence interferes with key developmental tasks of this period of life (e.g., graduating from school, vocational training, and finding a romantic partner), increasing the risk of deficits in psychosocial functioning in the long-term [4]. Therefore, adolescence represents a critical window of opportunity for early detection and intervention for BPD [3, 5]. Although controversial in the past, there is now convincing evidence that BPD is a reliable and valid diagnosis in adolescence, and that early intervention can lead to meaningful improvements in adolescents with BPD features or a first manifestation of full-threshold BPD [6, 7].

Current clinical guidelines for BPD recommend outpatient psychotherapy as the first-line treatment [8–11]. Inpatient care is only recommended for short-term crisis intervention for patients at high risk of suicide or in the case of specialized psychotherapy programs (e.g., dialectical behaviour therapy, DBT) adapted for the inpatient setting. In any case, inpatient treatment should be clearly time-limited and goal-oriented, with longer inpatient stays being assumed to have the potential to further exacerbate symptoms (e.g., self-harming behaviour) and increase functional deficits, promoting long-term dependency on the healthcare system [12]. However, treatment reality looks alarmingly different for most patients with BPD, in particular for young people: a lack of outpatient services that provide low-threshold access and evidence-based diagnosis and treatment, non-guideline-based treatment with lengthy, poorly goal-oriented inpatient stays and ineffective or even harmful polypharmacy, overly complex and (with regard to place, time, and delivery mode) inflexible treatments that do not meet the needs and preferences of young people, and a disruption of care in the transition from the child and adolescent to the adult mental health care system [12, 13].

If one consults the current clinical guidelines for BPD on the question of the most appropriate treatment setting [8–11], two things stand out: first, most current clinical guidelines for BPD focus on adults and only marginally address adolescents, if at all; second, the recommendation of outpatient psychotherapy as first-line treatment seems to be based rather on consensus than empirical evidence. The reason for this lies in a dearth of research on the question of the appropriate treatment setting for people with BPD in general, and for young people with BPD features in particular. In a recent systematic review on psychological interventions for people with BPD [14], 63 out of 75 included trials were performed in outpatient settings, five in inpatient settings, and seven in both in- and outpatient settings. Outpatient treatment was found to be more effective in reducing BPD symptom severity than inpatient treatment, while inpatient treatment was found to be more effective in improving psychosocial functioning than outpatient treatment. With the majority of studies included in the review being conducted on adults with BPD (only 3 of the 75 included studies focused on adolescents), little is known about the effect of the treatment setting (in- vs. outpatient) on the outcome of early intervention for adolescents with BPD pathology.

To close this gap, the current study examined whether adolescents receiving only outpatient treatment differ from those receiving combined outpatient/inpatient treatment with regard to changes in symptomatology, psychosocial functioning, and quality of life over a 2-year follow-up. In accordance with the recommendation in current clinical guidelines for BPD [8–11], we hypothesized that adolescent outpatients would show greater improvements in symptomatology, psychosocial functioning, and quality of life over time compared to adolescent inpatients.

## Methods

### Participants and procedure

Participants were consecutively recruited from a specialized outpatient clinic for early intervention for BPD (AtR!Sk; *Ambulanz für Risikoverhalten und Selbstschädigung* [15] at the Clinic for Child and Adolescent Psychiatry in Heidelberg, Germany. AtR!Sk is aimed at adolescents aged 12–17 years who display risk-taking or self-harm behaviours. Patients who underwent the diagnostic assessment were offered either (i) treatment in AtR!Sk, or (ii) treatment in the public healthcare system outside of AtR!Sk [15]. Treatment in AtR!Sk is highly standardized and includes short-term cognitive-behavioural therapy particularly addressing (non-suicidal) self-injury (i.e., the Cutting Down Program) [16] and/or Dialectical Behavioural Therapy for Adolescents (DBT-A) [17] according to a stepped-care approach, along with psychiatric management and specialist crisis involvement (e.g., outpatient crisis interventions or time-limited admission to the acute ward) when necessary. A substantial proportion of patients yearned for inpatient treatment, e.g., to escape aversive living situations or due to living too far away for weekly outpatient treatment sessions in AtR!Sk. The inpatient treatment comprised of a less-standardized but multimodal, interdisciplinary treatment that was conducted in accordance with the DBT-A principles. In addition to this, both DBT-A informed individual psychotherapy and skills group were conducted weekly with the patients.

The AtR!Sk cohort study was approved by the Ethical Committee of the Medical Faculty, Heidelberg University, Germany (Study: ID S-449/2013). Baseline assessments were conducted between 2013 and 2020. Inclusion criteria were: 12–17 years of age and participation in the AtR!Sk diagnostic phase. Exclusion criteria were: insufficient German language skills; impairment of intellectual functioning; and diagnosis of bipolar disorder, schizophrenia or schizoaffective disorder. Written informed consent (or assent, respectively) was obtained from all participants, and also from a parent or legal guardian for those under the age of 16 years. Initial baseline assessments were part of the usual diagnostic procedure of the AtR!Sk clinic. Further assessments were conducted 1 year after baseline (follow-up 1) and 2 years after baseline (follow-up 2). Participants received 20 Euros for each follow-up interview.

In the present analysis, only data from participants who attended the two follow-up assessments and received any psychiatric/psychotherapeutic treatment during the first year after baseline were included. Participants were divided into two groups depending on whether they had received inpatient treatment during the first year that lasted 8 days or longer (i.e., combined inpatient/outpatient group) or not (i.e., outpatient only group). The group allocation was based on the following rationale: outpatient treatment for people with BPD should include the possibility for inpatient crisis intervention in the case of acute suicidality. Inpatient crisis intervention usually aims to reduce potential damage to an individual and lasts no longer than a few days. In contrast, inpatient stays lasting longer than a week probably go beyond the mere survival and stabilization of the affected person and can, thus, be considered as more comprehensive inpatient treatments.

### Measures

Sociodemographic information, including age, sex, and type of school, was assessed in a standardized way.

Psychiatric disorders were assessed at baseline using the Mini-International Neuropsychiatric Interview for Children and Adolescents (MINI-KID 6.0) [18]. The M.I.N.I.-Kid is a short, structured interview to assess psychiatric diagnoses according to the DSM-IV and ICD-10, with good psychometric properties [18, 19].

The number of BPD criteria fulfilled served as a proxy of BPD severity. The nine DSM-IV BPD criteria were assessed using the respective section of the Structured Clinical Interview for DSM-IV axis II (SCID-II) [20]. Each item is scored on a three-point scale (1 = absent, 2 = sub-threshold, and 3 = present). A BPD criteria is scored present for under-age individuals if it has been present for at least 1 year during most of the time [21]. The diagnosis requires that five or more criteria are met.

Psychosocial functioning was assessed with the Global Assessment of Functioning (GAF) Scale [22]. It is an observer-rated overall measure of psychological, social, and occupational functioning that covers the range from severe psychopathology (score of 1) to positive mental health (score of 100).

The Self-Injurious Thoughts and Behaviours Interview (SITBI) [23] was applied to assess the number of days with non-suicidal self-injury (NSSI), the number of days with suicidal thoughts, and the number of suicide attempts during the last 12 months.

The Depression Inventory for Children and Adolescents (German translation: Depressionsinventar für Kinder und Jugendliche; DIKJ) [24] was administered to assess severity of depressive symptoms. It is a self-report measure that consists of 27 items rated on a three-point scale (0 = symptom is not present, 1 = symptom is present with medium severity, and 2 = symptom is present with strong severity). The sum score was used for the current analysis.

Psychopathological distress was assessed using the Symptom-Checklist-90 Revised (SCL-90-R) [25]; a self-report measure consisting of 90 items that are rated on a five-point scale, ranging from 0 (not at all) to 4 (very much). The SCL-R-90 Global Severity Index (SCL-GSI) is built by the mean value of all items and used as a measure of overall psychopathological distress in the current study.

Health-related quality of life was measured using the KIDSCREEN-10 [26]. The KIDSCREEN-10 is a self-report measure, consisting of 10 items that are scored on a five-point scale, ranging from 1 (not at all/never) to 5 (extremely/never). The sum score was used in the current analysis.

The Clinical Global Impressions Scale-Severity (CGIs) [27] is an observer-rated global measure of illness severity within the past 7 days that ranges from 1 (not ill at all) to 7 (severely ill).

### Data analysis

To check for a systematic loss of participants, participants included in the current analysis were compared with those who were not with regard to age, sex, school type, ICD-10 diagnoses, and number of BPD criteria at baseline, using stepwise logistic regression, minimizing Bayes Information Criterion.

Differences between the two groups in sociodemographic variables, psychiatric diagnoses, number of outpatient treatment sessions, number of days of inpatient treatment, and number of BPD criteria were tested using one-way analyses of variance (ANOVA) or Wilcoxon rank-sum tests for continuous variables and Chi-square (*χ*^*2*^) or Fisher’s exact tests for categorical variables.

Missing value analysis is presented in the Supplementary Materials (SM). Multiple imputation using chained equations was applied under the assumption of missing at random to impute missing data. Predictive mean matching using the five nearest neighbours was used to perform 20 imputations. Age and sex at baseline were used to start the imputation [28].

To examine the hypothesis that the outpatient only group would show greater clinical improvements over time compared to the combined inpatient/outpatient group, a series of multilevel models was conducted based on the imputed data. As the study was non-randomized, inverted probability weights were used to adjust for initial baseline differences in patient characteristics between the combined inpatient/outpatient group and the outpatient only group in all models [29]. The inverted probability weights procedure is described in more detail in the SM. To estimate the impact of group and time on the outcome variables, multilevel mixed-effects linear regressions were conducted. There were a few exceptions: multilevel generalized linear models were calculated for the number of fulfilled BPD criteria (model with binomial distribution with nine trials), the number of days with suicidal ideation or NSSI, and the number of suicide attempts in the past 12 months (models with negative binomial distribution) as outcome variables. Each generalized linear or mixed model contained a fixed effect for group (combined inpatient/outpatient group, outpatient only group), time (baseline, follow-up 1, and follow-up 2), and for the group-by-time interaction. Observations were grouped by subject ID. Significant main effects (group, time, group-by-time interaction) were followed by post hoc contrasts, using the Wald test. In the case of a significant interaction effect, two kinds of contrasts were conducted to (1) compare changes in outcome variables over time between groups, and (2) to compare outcome variables at each time point between groups. *p* values were adjusted for false discovery rate when performing multiple comparisons according to the method proposed by Benjamini-Hochberg [30].

Two sensitivity analyses were conducted: All models were reanalysed (A) based on the raw data of all participants without the use of inversed probability weights, and (B) based on the raw data of only those participants who had no missing values with inversed probability weights. The latter models did not include all participants, as it was not possible to calculate inversed probability weights for participants with missing values. The main results based on imputed data and adjusted for baseline differences between participants are presented below; the results of the sensitivity analyses are presented in the SM (Tables 2 and 3).

All analyses were conducted in Stata/SE (17.0, Stata Corp LLC, College Station, TX, USA). The significance level was set as *α* = 0.05.

## Results

### Sample characteristics

Of 782 adolescents who presented at the specialized outpatient clinic, a total of *n* = 673 participants completed the baseline assessment and provided written informed consent to participate in the longitudinal AtR!Sk cohort study (participation rate of 86%). Of the 673 participants, 348 (52% of the sample at baseline) were assessed at follow-up 1 and 260 (75% of the sample at follow-up 1) were assessed at follow-up 2. In the present analysis, only data from patients who attended both follow-up assessments (*n* = 220, 33% of the sample at baseline) and received any psychiatric/psychotherapeutic treatment during the first year after baseline (*n* = 178, 81% of participants who had attended both follow-ups) were included. Of the *n* = 178 adolescents, 63 participants were assigned to the combined inpatient/outpatient group and 115 participants to the outpatient only group.

Participants included in the current analysis (*n* = 178) were significantly younger (Odds Ratio [OR] = 1.18, 95% Confidence Interval [CI] = 1.04–1.34, *p* = 0.010), had more BPD criteria (OR = 0.82, 95%CI = 0.76–0.89, *p* < 0.001), and fewer ICD-10 F9 diagnoses (OR = 2.60, 95%CI =1.69–4.00, *p* < 0.001) compared to those who were excluded (*n* = 495). No significant group differences were found with regard to sex, school type, and ICD-10 F1-F8 diagnoses.

Sociodemographic and clinical characteristics of the combined inpatient/outpatient group and the outpatient only group at baseline are provided in Table 1. There were no significant differences between the two groups, with the exception of behavioural syndromes associated with physiological disturbances and physical factors (ICD-10 F5) that were more common in the combined inpatient/outpatient group than in the outpatient only group. The combined inpatient/outpatient group received significantly more outpatient sessions (first year M = 17.51 (SD = 17.99), *p* = 0.007; second year M = 29.27 (SD = 30.78), *p* = 0.004) and had significantly more days of inpatient treatment (first year M = 58.27 (SD = 58.53), *p* < 0.001; second year M = 19.38 (SD = 50.88), *p* = 0.005) compared with the outpatient only group (outpatient sessions: first year M = 23.42 (SD = 16.71); second year M = 17.55 (SD = 22.15); days of inpatient treatment: first year M = 0.85 (SD = 2.27); second year M = 3.94 (SD = 18.46)) in both follow-up years. Means and standard deviations of each outcome per group and time point are provided in the SM (Table 1).

**Table 1:** Sociodemographic and clinical characteristics of the sample at baseline (*N* = 178)

	Combined inpatient/outpatient group (*n* = 63)	Outpatient only group (*n* = 115)	*p*
Age (years), mean (SD)	15.17 (1.41)	14.73 (1.49)	0.055
Female gender, *n* (%)	55 (87%)	108 (94%)	0.129
Type of school^1^, *n* (%)			0.546
Hauptschule	2 (3%)	8 (7%)	
Realschule	17 (27%)	37 (32%)	
Gymnasium	32 (51%)	51 (45%)	
Other school	8 (13%)	15 (12%)	
Not attending school	4 (6%)	3 (3%)	
Psychiatric diagnoses according to ICD-10^2^, *n* (%)			
Substance use disorders (F1)	10 (16%)	24 (21%)	0.417
Schizophrenia, schizotypal and delusional disorders (F2)	0 (0%)	0 (0%)	–
Affective disorders (F3)	47 (75%)	81 (70%)	0.554
Neurotic, stress-related somatoform disorders (F4)	21 (33%)	49 (43%)	0.226
Behavioural syndromes associated with physiological disturbances and physical factors (F5)	18 (29%)	10 (9%)	< 0.001
Disorders of adult personality and behaviour (F6)	29 (46%)	61 (53%)	0.371
Mental retardation (F7)	0 (0%)	0 (0%)	–
Disorders of psychological development (F8)	0 (0%)	0 (0%)	–
Behavioural and emotional disorders with onset usually occurring in childhood and adolescence (F9)	10 (16%)	24 (21%)	0.417
Number of BPD criteria met, mean (SD)	3.71 (2.11)	4.12 (2.21)	0.234
Number of individuals with at least 5 BPD criteria met, *n* (%)	21 (33%)	53 (46%)	0.099

*p *values refer to group differences based on χ^2^ tests/Fisher’s exact tests (categorical variables) or one-way ANOVAs (continuous variables)

*BPD* borderline personality disorder, *ICD-10* International Statistical Classification of Diseases and Related Health Problems, 10th revision

^1^School types: After 4 years of elementary school the German school system branches into three types of secondary schools. The so-called Hauptschule (Secondary General School, which takes 5 years after Primary School) prepares pupils for vocational training, whereas the Realschule (Intermediate Secondary School) concludes with a general certificate of secondary education after 6 years. Eight years of Gymnasium provide pupils with a general university entrance qualification

^2^Multiple diagnoses possible

### Differential treatment courses

**Fig. 1:**
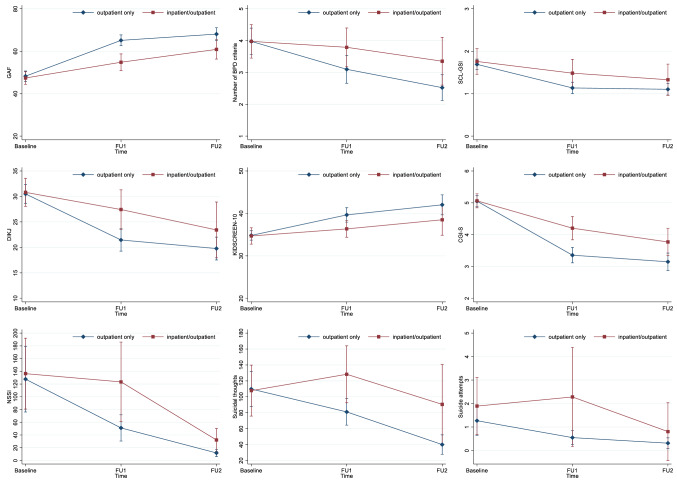
Differential trajectories of the outcome variables between baseline and follow-up at 12 (FU1) and 24 months (FU2), respectively, for the combined inpatient/outpatient group and the outpatient only group. *BPD* borderline personality disorder, *CGI-S* Clinical Global Impression Scale-Severity, *DIKJ* Depression Inventory for Children and Adolescents, *GAF* Global Assessment of Functioning, *NSSI* non-suicidal self-injury, *SCL-GSI* Symptom-Checklist-90 Revised, *SITBI* self-injurious thoughts and behaviours interview

Detailed results of the generalized and mixed models predicting the outcome variables are presented in Table 2 and illustrated in Fig. 1.

**Table 2:** Results of generalized and mixed models

Outcome	Model fit	Main effects		Contrasts					
		Predictors		Predictors	β	SE	95% CI	*p* value	*p* value adjusted for multiple comparisons
BPD (number of criteria)	*χ*^2^(5) = 54.39, *p* < 0.001	group	*χ*^2^ (1) = 2.22, *p* = 0.136						
		time	*χ*^2^ (2) = 14.93, *p* = 0.001	FU1 vs. baseline	− 0.27	0.09	− 0.45, − 0.08	0.005	0.010
				FU2 vs. FU1	− 0.27	0.10	− 0.46, − 0.08	0.005	0.011
				FU2 vs. baseline	− 0.53	0.14	− 0.80, − 0.26	< 0.001	< 0.001
		(group)#(time)	*χ*^2^ (2) = 4.28, *p* = 0.118						
GAF	*χ*^2^(5) = 158.93–211.35, *p* < 0.001	group	*χ*^2^ (1) = 12.74, *p* < 0.001	Inpatient/outpatient vs. outpatient	− 6.39	1.79	− 9.90, − 2.88	< 0.001	0.001
		time	*χ*^2^ (2) = 87.29, *p* < 0.001	FU1 vs. baseline	10.97	1.45	8.13, 13.82	< 0.001	< 0.001
				FU2 vs. FU1	3.97	1.27	1.47, 6.47	0.002	0.005
				FU2 vs. baseline	14.95	1.67	11.68, 18.21	< 0.001	< 0.001
		(group)#(time)	*χ*^2^ (2) = 13.03, *p* = 0.001	(FU1 vs. baseline)#(inpatient/outpatient vs. outpatient)	− 10.34	2.91	− 16.04, − 4.64	< 0.001	0.001
				(FU2 vs. FU1)#(inpatient/outpatient vs. outpatient)	3.45	2.54	− 1.53, 8.44	0.175	0.217
				(FU2 vs. baseline)#(inpatient/outpatient vs. outpatient)	− 6.89	3.33	− 13.42, − 0.35	0.039	0.053
			group@time	(inpatient/outpatient vs. outpatient) @baseline	− 0.65	2.00	− 4.58, 3.28	0.745	0.772
				(inpatient/outpatient vs. outpatient)@FU1	− 10.99	2.36	− 15.61, − 6.37	< 0.001	< 0.001
				(inpatient/outpatient vs. outpatient) @FU2	− 7.54	2.96	− 13.33, − 1.74	0.011	0.020
NSSI (number of days in the past 12 months; SITBI)	*χ*^2^(5) = 133.22, *p* < 0.001	group	*χ*^2^ (1) = 4.68, *p* = 0.031	inpatient/outpatient vs. outpatient	0.59	0.27	0.06, 1.13	0.031	0.046
		time	*χ*^2^ (2) = 111.27, *p* < 0.001	FU1 vs. baseline	− 0.52	0.14	− 0.79, − 0.25	< 0.001	0.001
				FU2 vs. FU1	− 1.42	0.17	− 1.76, − 1.08	< 0.001	< 0.001
				FU2 vs. baseline	− 1.94	0.18	− 2.30, − 1.58	< 0.001	< 0.001
		(group)#(time)	*χ*^2^ (2) = 10.54, *p* = 0.005	(FU1 vs. baseline)#(inpatient/outpatient vs. outpatient)	0.80	0.27	0.27, 1.33	0.003	0.007
				(FU2 vs. FU1)#(inpatient/outpatient vs. outpatient)	0.09	0.35	− 0.59, 0.78	0.786	0.800
				(FU2 vs. baseline)#(inpatient/outpatient vs. outpatient)	0.90	0.36	0.19, 1.61	0.014	0.024
			group@time	(inpatient/outpatient vs. outpatient) @baseline	0.03	0.26	− 0.49, 0.54	0.923	0.923
				(inpatient/outpatient vs. outpatient) @FU1	0.83	0.33	0.17, 1.48	0.014	0.023
				(inpatient/outpatient vs. outpatient) @FU2	0.92	0.39	0.16, 1.69	0.018	0.029
Suicidal thoughts (number of days in the past 12 months; SITBI)	*χ*^2^(5) = 41.12, *p* < 0.001	group	*χ*^2^ (1) = 7.06, *p* = 0.008	Inpatient/outpatient vs. outpatient	0.43	0.16	0.11, 0.74	0.008	0.016
		time	*χ*^2^ (2) = 12.35, *p* = 0.002	FU1 vs. baseline	− 0.05	0.13	− 0.30, 0.20	0.690	0.743
				FU2 vs. FU1	− 0.50	0.14	− 0.77, − 0.22	< 0.001	0.001
				FU2 vs. baseline	− 0.55	0.20	− 0.94, -0.16	0.006	0.012
		(group)#(time)	*χ*^2^ (2) = 5.18, *p* = 0.075						
Suicide attempts (number in the past 12 months; SITBI)	*χ*^2^(5) = 29.71–49.04, *p* < 0.001	group	*χ*^2^ (1) = 3.94, *p* = 0.047	inpatient/outpatient vs. outpatient	0.87	0.44	0.01, 1.73	0.047	0.061
		time	*χ*^2^ (2) = 9.15, *p* = 0.010	FU1 vs. baseline	− 0.26	0.26	− 0.78, 0.25	0.311	0.363
				FU2 vs. FU1	− 0.77	0.26	− 1.28, − 0.25	0.004	0.009
				FU2 vs. baseline	− 1.03	0.39	− 1.80, − 0.26	0.009	0.017
		(group)#(time)	*χ*^2^ (2) = 5.30, *p* = 0.071						
DIKJ	*χ*^2^(5) = 107.19–145.04, *p* < 0.001	group	*χ*^2^ (1) = 3.37, *p* = 0.066						
		time	*χ*^2^ (2) = 40.76, *p* < 0.001	FU1 vs. baseline	− 6.74	1.11	− 8.91, − 4.57	< 0.001	< 0.001
				FU2 vs. FU1	− 2.31	0.97	− 4.22, − 0.40	0.018	0.030
				FU2 vs. baseline	− 9.05	1.40	− 11.79, − 6.30	< 0.001	< 0.001
		(group)#(time)	*χ*^2^ (2) = 6.34, *p* = 0.042	(FU1 vs. baseline)#(inpatient/outpatient vs. outpatient)	4.65	2.20	0.34, 8.97	0.035	0.051
				(FU2 vs. FU1)#(inpatient/outpatient vs. outpatient)	− 2.72	1.81	− 6.27, 0.82	0.132	0.168
				(FU2 vs. baseline)#(inpatient/outpatient vs. outpatient)	1.93	2.73	− 3.42, − 7.28	0.480	0.538
			group@time	(inpatient/outpatient vs. outpatient) @baseline	1.03	1.65	− 2.20, 4.27	0.531	0.583
				(inpatient/outpatient vs. outpatient) @FU1	5.69	2.26	1.27, 10.11	0.012	0.021
				(inpatient/outpatient vs. outpatient)@FU2	2.96	2.58	− 2.09, 8.02	0.251	0.299
SCL-GSI	*χ*^2^(5) = 105.37–122.01, *p* < 0.001	group	*χ*^2^ (1) = 1.66, *p* = 0.198						
		time	*χ*^2^ (2) = 61.19, *p* < 0.001	FU1 vs. baseline	− 0.45	0.06	− 0.58, − 0.33	< 0.001	< 0.001
				FU2 vs. FU1	− 0.07	0.06	− 0.18, − 0.04	0.214	0.261
				FU2 vs. baseline	− 0.52	0.07	− 0.66, − 0.38	< 0.001	< 0.001
		(group)#(time)	*χ*^2^ (2) = 2.96, *p* = 0.228						
KIDSCREEN-10	*χ*^2^(5) = 61.60–90.38, *p* < 0.001	group	*χ*^2^ (1) = 2.54, *p* = 0.111						
		time	*χ*^2^ (2) = 40.73, *p* < 0.001	FU1 vs. baseline	3.93	0.70	2.55, 5.31	< 0.001	< 0.001
				FU2 vs. FU1	1.53	0.73	0.09, 2.97	0.037	0.052
				FU2 vs. baseline	5.46	0.87	3.75, 7.17	< 0.001	< 0.001
		(group)#(time)	*χ*^2^ (2) = 2.34, *p* = 0.311						
CGI-S	*χ*^2^(5) = 209.69–278.57, *p* < 0.001	group	*χ*^2^ (1) = 9.18, *p* = 0.002	inpatient/outpatient vs. outpatient	0.52	0.17	0.18, 0.85	0.002	0.007
		time	*χ*^2^ (2) = 118.19, *p* < 0.001	FU1 vs. baseline	− 1.24	0.13	− 1.50, − 0.98	< 0.001	< 0.001
				FU2 vs. FU1	− 0.24	0.12	− 0.47, − 0.02	0.035	0.050
				FU2 vs. baseline	− 1.48	0.14	− 1.77, − 1.20	< 0.001	< 0.001
		(group)#(time)	*χ*^2^ (2) = 8.83, *p* = 0.012	(FU1 vs. baseline)#(inpatient/outpatient vs. outpatient)	0.78	0.26	0.27, 1.30	0.003	0.007
				(FU2 vs. FU1)#(inpatient/outpatient vs. outpatient)	− 0.19	0.23	− 0.64, 0.27	0.423	0.484
				(FU2 vs. baseline)#(inpatient/outpatient vs. outpatient)	0.60	0.29	0.02, 1.17	0.042	0.056
			group@time	(inpatient/outpatient vs. outpatient) @baseline	0.05	0.15	− 0.24, 0.35	0.712	0.752
				(inpatient/outpatient vs. outpatient) @FU1	0.84	0.24	0.37, 1.30	< 0.001	< 0.001
				(inpatient/outpatient vs. outpatient)@FU2	0.65	0.28	0.10, 1.20	0.020	0.031

*p* values adjusted for multiple comparison according to Benjamini–Hochberg

*BPD* borderline personality disorder, *CGI-S* Clinical Global Impression Scale-Severity, *DIKJ* Depression Inventory for Children and Adolescents, *FU1* follow-up 1 12 months after baseline, *FU2*: follow-up 2 24 months after baseline, *GAF* Global Assessment of Functioning, *NSSI* non-suicidal self-injury, *SCL-GSI* Symptom-Checklist-90 Revised, *SITBI* self-injurious thoughts and behaviours interview

The main effect of time was significant in all models. There was a significant reduction of the number of BPD criteria, the number of days with NSSI, and depressive symptoms, as well as a significant increase in psychosocial functioning from baseline to follow-up 1 and from follow-up 1 to follow-up 2. Psychopathological distress, severity of illness, and quality of life significantly decreased between baseline and follow-up 1, with no further improvement in the later course. Suicidal thoughts and attempts significantly declined between follow-up 1 and follow-up 2, with no significant change early in the course.

In addition, there was a significant group-by-time interaction for psychosocial functioning, number of days with NSSI, depressive symptoms, and severity of illness. Contrasts revealed a significantly greater reduction in the number of days with NSSI and the severity of illness as well as a significant greater improvement in psychosocial functioning between baseline and follow-up 1 for the outpatient only group compared with the combined inpatient/outpatient group. In addition, the outpatient only group showed significantly fewer depressive symptoms at follow-up 1, and significantly fewer days with NSSI, lower severity of illness, and better psychosocial functioning at both, follow-up 1 and follow-up 2 compared with the combined inpatient/outpatient group.

## Discussion

Using data of a clinical cohort, this study examined differential clinical outcomes over a 2-year follow-up period of adolescents with BPD pathology receiving either exclusively outpatient treatment or combined inpatient and outpatient treatment. Two main findings emerged from the study.

First, there were significant clinical improvements over the 2-year follow-up period in both the combined inpatient/outpatient group and the outpatient only group, as indicated by a decrease in BPD features, depressive symptoms, psychopathological distress, days with NSSI or suicidal thoughts and suicide attempts within the past 12 months, and overall illness severity ratings, as well as by an increase in quality of life and psychosocial functioning. This finding contributes to the growing evidence that early intervention for adolescents with BPD features is feasible and efficient [3, 13]. Interestingly, while, for most outcomes, the improvement occurred continuously over the 2 years, there were a few remarkable exceptions. First, psychopathological distress, severity of illness, and quality of life showed a significant improvement in the first year, with no further change in the second year. Second, suicidal thoughts and attempts significantly decreased in the second year, with no change in the first year. This delayed improvement may be explained by the fact that suicidal thoughts and behaviour can serve as an avoidance or escape strategy that often persists for a long time and is difficult to change [31, 32].

Second, regarding differential effects, the outpatient only group showed a faster improvement in some outcome variables compared with the combined inpatient/outpatient group, as indicated by a greater decrease in the number of days with NSSI within the past 12 months and overall illness severity ratings as well as a greater increase in psychosocial functioning from baseline to follow-up 1, with comparable improvements from follow-up 1 to follow-up 2 between groups. The outpatient only group showed significant better psychosocial functioning, fewer days with NSSI, and lower severity of illness at both, follow-up 1 and follow-up 2, compared with the combined inpatient/outpatient group. Remarkably, the outpatient only group showed faster clinical improvements, even though the received significantly fewer outpatient sessions compared with the combined outpatient/inpatient group. As the analyses were adjusted for baseline differences in the outcome variables between participants, it is unlikely that the group differences in clinical improvements over time resulted from differences in severity of psychopathology or functional impairments at the beginning of treatment. However, due to the study design—which was an observational cohort study and not a randomized-controlled trial—we cannot rule out that other unknown factors have contributed to group differences in clinical improvements. Keeping this in mind, we tentatively interpret the results as indicating that both outpatient and inpatient treatment for adolescents with BPD features can be effective, but clinical improvements may be achieved somewhat faster in the outpatient setting than in the inpatient setting.

### Clinical implications and future research directions

Overall, the study results support specialized outpatient psychotherapy as first-line treatment for adolescents with BPD pathology and is, thus, in line with most current clinical guidelines for BPD that primarily address adults [8–11]. Specialized outpatient treatment for people with BPD has also been demonstrated to be a cost-effective option compared to the generally costly residential care [33, 34]. However, on the individual level, the clinical decision for a specific treatment setting should always be based on a careful evaluation of potential benefits and risks. While there are probably no absolute indications for inpatient treatment for adolescents with BPD, potential reasons in favour include the need for a comprehensive diagnostic assessment in a protected environment not feasible in the outpatient setting (e.g., because of drug and alcohol misuse), severe psychopathology that makes everyday activities (e.g., school attendance) difficult or impossible, risk of self-harm, or poor physical health requiring medical care (e.g., in the context of co-occurring eating disorders). On the other hand, potential reasons against inpatient treatment can be the required dislocation from routine everyday life, loss of family, friends, or community support, education disruption, stigma, and the acquisition of unhelpful or destructive behaviours learnt from inpatient peers. Additionally, contextual factors, such as the availability of specialized psychotherapy programs in the in- or outpatient setting, the availability of post-discharge services in the community, and family burden and preferences, should be taken into account in the shared decision-making process between clinicians, young people, and their families [12]. If inpatient treatment is considered as an option, it should be clear to all parties involved (i.e., adolescents, relatives, and clinicians) before admission that it is time-limited and oriented toward jointly predefined goals. Future research is needed to investigate the role of inpatient treatment within stepped-care models for adolescents with BPD pathology [6, 35] and home treatment as an alternative to inpatient treatment [36].

### Limitations

There are several limitations to be considered. First, the representativeness of our findings is limited, because participants who were excluded from the current analysis were older, had less BPD criteria, and more ICD-10 F9 diagnoses. Second, there were no “pure” groups: both the outpatient only group and the combined inpatient/outpatient group received outpatient treatment of comparable intensity, and in both groups, shorter inpatient stays (≤ 7 days) were possible. Third, while the outpatient treatment consisted of manualized and regularly supervised brief CBT [16] or DBT-A [17], the inpatient treatment also followed DBT-A principles, but was less-standardized. Thus, the findings in favour of the outpatient only group over the combined inpatient/outpatient group may be due to a greater specialization of the outpatient treatment compared with the inpatient treatment in the current study. Forth, as group assignment was not randomized, the analyses were adjusted for individual differences at baseline. However, the decision for inpatient treatment was not necessarily made at baseline, but could have taken place anytime later in the first year. Therefore, we cannot rule out that individual differences in the outcome variables at the time point of the decision have influenced the results. In addition, group differences in other factors that may have influenced the decision for inpatient treatment (e.g., lower self-efficacy, self-reliance, or social support) could have contributed to the less favourable treatment outcomes for the combined outpatient/inpatient group. Future studies applying a randomized-controlled design are required to address this limitation. Fifth, treatments that the participants received between follow-up 1 and follow-up 2 were not considered as covariates in the statistical analyses. However, as the combined inpatient/outpatient group received significantly more outpatient sessions and had more days of inpatient treatment compared with the outpatient only group, it seems unlikely that the faster improvement observed in some clinical outcomes in the latter group is due to a more intense treatment during this time period.

## Conclusion

In the current study on adolescents with BPD pathology, both outpatient treatment alone and the combination of outpatient and inpatient treatment resulted in clinical improvements over the 2-year follow-up period, with some indications for faster improvements in those patients who received only outpatient treatment. The findings support early intervention efforts for young people with sub-threshold or first manifestation BPD and provide preliminary evidence that the recommendation of outpatient psychotherapy as the first-line treatment inherent in most current clinical guidelines for (adults with) BPD also holds true for adolescents, even though a careful weighing of the potential advantages and disadvantages of inpatient treatment in each individual case is indispensable.

### Supplementary Information

Below is the link to the electronic supplementary material.

Supplementary file1 (DOCX 48 KB)

## Data Availability

Data are available upon request from the corresponding author.
