# Supplementary material for: Differential outcomes of outpatient only versus combined inpatient/outpatient treatment in early intervention for adolescent borderline personality disorder
Source: Eur Child Adolesc Psychiatry. 2023 May 11;33(4):1005–16. doi: 10.1007/s00787-023-02222-8 (PMC11032290; doi:10.1007/s00787-023-02222-8)
Supplement: Supplementary file 1 — Supplementary file1 (DOCX 48 KB) [file 787_2023_2222_MOESM1_ESM.docx]

**Supplementary Materials for:**

**Differential outcomes of outpatient only versus combined inpatient/outpatient treatment**

**in early intervention for adolescent borderline personality disorder**

This includes:

- Missing value analysis
- Inverted probability weights procedure
- Table 1: Means and standard deviations of the outcome variables at baseline, follow-up 1, and follow-up 2 for the combined inpatient/outpatient and the outpatient only group, based on imputed data
- Table 2: Sensitivity analysis A (Generalized and mixed models based on raw data of all participants without the use of inverted probability weights to adjust for initial differences in patient characteristics)
- Table 3: Sensitivity analysis B (Generalized and mixed models based on raw data of only those participants who had no missing values with inverted probability weights to adjust for initial differences in patient characteristics)

**Missing value analysis**

Missing value analysis revealed incomplete data in the following variables: psychosocial functioning (GAF; 8.1% missing), number of suicide attempts during the last 12 months (SITBI; 1.5% missing). depressive symptoms (DIKJ; 17.4% missing), psychological distress (SCL-GSI; 3.7% missing), quality of life (KIDSCREEN-10; 25.8% missing), and severity of illness (CGI-S; 4.3% missing).

**Inverted probability weights**

This method involves two steps. First, a propensity score representing the probability that a patient received inpatient treatment was calculated for each patient based on baseline variables that may have guided the clinical decision regarding the treatment setting: sex, age, ICD-F5 diagnosis, number of BPD criteria (SCID-II), psychosocial functioning (GAF), number of days with suicidal ideation or NSSI and number of suicide attempts during the past 12 months (SITBI), depressive symptoms (DIKJ), psychological distress (SCL-GSI), quality of life (KIDSCREEN-10), and severity of illness (CGI-S). Second, the probability score was inverted and then used to weight each patient. For the combined inpatient/outpatient group, the weight was calculated as 1/propensity score, whereas for the outpatient only group it was calculated as 1/(1 - propensity score).

***Table 1****.* Means and standard deviations of the outcome variables at baseline, follow-up 1, and follow-up 2 for the combined inpatient/outpatient group and the outpatient only group, based on non-imputed data (*N* = 178).

| **Outcome** | **Baseline** | | **Follow-up 1** | | **Follow-up 2** | |
| --- | --- | --- | --- | --- | --- | --- |
| Group | Combined inpatient/outpatient group  (n = 63) | Outpatient only group (n = 115) | Combined inpatient/outpatient group (n = 63) | Outpatient only group (n = 115) | Combined inpatient/outpatient group (n = 63) | Outpatient only group (n = 115) |
| BPD (number of criteria) | 3.71 (2.11) | 4.12 (2.21) | 3.60 (1.91) | 3.25 (2.28) | 3.05 (2.21) | 2.64 (2.07) |
| GAF | 45.26 (10.13) | 48.85 (12.05) | 54.97 (12.25) | 65.02 (14.07) | 60.37 (17.16) | 67.24 (15.92) |
| NSSI (number of days in the past 12 months; SITBI) | 68.60 (76.15) | 79.61 (84.34) | 70.73 (80.17) | 40.78 (57.01) | 31.67 (57.86) | 13.63 (29.67) |
| Suicidal thoughts (number of days in the past 12 months; SITBI) | 125.79 (119.58) | 101.40 (107.24) | 119.11 (97.00) | 80.24 (88.53) | 77.54 (116.95) | 41.52 (67.42) |
| Suicide attempts (number in the past 12 months; SITBI) | 0.94 (1.45) | 1.43 (5.46) | 1.19 (2.53) | 0.52 (1.43) | 0.35 (1.48) | 0.39 (1.92) |
| DIKJ | 30.88 (9.65) | 30.63 (9.50) | 26.90 (10.43) | 22.17 (11.28) | 22.47 (11.86) | 19.98 (10.53) |
| SCL-GSI | 1.66 (0.72) | 1.72 (0.66) | 1.36 (0.73) | 1.17 (0.76) | 1.19 (0.76) | 1.14 (0.71) |
| KIDSCREEN-10 | 34.37 (6.83) | 34.44 (5.66) | 36.70 (6.02) | 39.32 (8.24) | 39.56 (7.45) | 41.79 (10.37) |
| CGI-S | 5.33 (0.81) | 4.94 (0.93) | 4.14 (1.26) | 3.38 (1.31) | 3.76 (1.53) | 3.21 (1.45) |

*Notes.* BPD, borderline personality disorder; CGI-S: Clinical Global Impression Scale-Severity; DIKJ, Depression Inventory for Children and Adolescents; GAF, Global Assessment of Functioning; NSSI: non-suicidal self-injury; SCL-GSI, Symptom-Checklist-90 Revised; SITBI, Self-Injurious Thoughts and Behaviours Interview.

***Table 2.*** Sensitivity analysis A (Generalized and mixed models based on raw data of all participants without the use of inverted probability weights to adjust for initial differences in patient characteristics)

| **Outcome** | **Modell fit** | **Main effects** | | **Contrasts** | | | | | |
| --- | --- | --- | --- | --- | --- | --- | --- | --- | --- |
|  |  | Predictors |  | Predictors | β | SE | 95% CI | p-value | p-value adjusted for multiple comparisons |
| BPD (number of criteria) | *χ^2^* (5) = 72.48, *p* < 0.001 | group | *χ^2^* (1) = 0.45, *p* = 0.502 |  |  |  |  |  |  |
|  |  | time | *χ^2^* (2) = 48.74, *p* < 0.001 | FU1 vs. baseline | -0.26 | 0.08 | -0.42, -0.11 | 0.001 | 0.003 |
|  |  |  |  | FU2 vs. FU1 | -0.32 | 0.08 | -0.48, -0.15 | < 0.001 | < 0.001 |
|  |  |  |  | FU2 vs. baseline | -0.58 | 0.08 | -0.74, -0.42 | < 0.001 | < 0.001 |
|  |  | (group)#(time) | *χ^2^* (2) = 9.73, *p* = 0.008 | (FU1 vs. baseline)#( inpatient/outpatient vs. outpatient) | 0.41 | 0.16 | 0.10, 0.72 | 0.010 | 0.017 |
|  |  |  |  | (FU2 vs. FU1)#( inpatient/outpatient vs. outpatient) | 0.05 | 0.17 | -0.28, 0.38 | 0.766 | 0.766 |
|  |  |  |  | (FU2 vs. baseline)#( inpatient/outpatient vs. outpatient) | 0.46 | 0.17 | 0.14, 0.78 | 0.005 | 0.009 |
|  |  |  | group@time | (inpatient/outpatient vs. outpatient)@baseline | -0.19 | 0.17 | -0.53, 0.14 | 0.264 | 0.296 |
|  |  |  |  | (inpatient/outpatient vs. outpatient)@FU1 | 0.22 | 0.17 | -0.12, 0.56 | 0.206 | 0.242 |
|  |  |  |  | (inpatient/outpatient vs. outpatient)@FU2 | 0.27 | 0.18 | -0.08, 0.62 | 0.132 | 0.169 |
| GAF | *χ^2^* (5) = 213.48, *p* < 0.001 | group | *χ^2^* (1) = 15.87, *p* < 0.001 | inpatient/outpatient vs. outpatient | -6.83 | 1.71 | -10.19, -3.47 | < 0.001 | < 0.001 |
|  |  | time | *χ^2^* (2) = 165.35, *p* < 0.001 | FU1 vs. baseline | 12.51 | 1.30 | 9.96, 15.06 | < 0.001 | < 0.001 |
|  |  |  |  | FU2 vs. FU1 | 3.81 | 1.23 | 1.41, 6.22 | 0.002 | 0.004 |
|  |  |  |  | FU2 vs. baseline | 16.32 | 1.31 | 13.75, 18.89 | < 0.001 | < 0.001 |
|  |  | (group)#(time) | *χ^2^* (2) = 6.19, *p* = 0.045 | (FU1 vs. baseline)#( inpatient/outpatient vs. outpatient) | -6.45 | 2.60 | -11.54, -1.35 | 0.013 | 0.019 |
|  |  |  |  | (FU2 vs. FU1)#( inpatient/outpatient vs. outpatient) | 3.22 | 2.45 | -1.59, 8.02 | 0.190 | 0.232 |
|  |  |  |  | (FU2 vs. baseline)#( inpatient/outpatient vs. outpatient) | -3.23 | 2.62 | -8.37, 1.91 | 0.218 | 0.250 |
|  |  |  | group@time | (inpatient/outpatient vs. outpatient)@baseline | -3.60 | 2.38 | -8.26, 1.06 | 0.130 | 0.170 |
|  |  |  |  | (inpatient/outpatient vs. outpatient)@FU1 | -10.05 | 2.19 | -14.34, -5.75 | < 0.001 | < 0.001 |
|  |  |  |  | (inpatient/outpatient vs. outpatient)@FU2 | -6.83 | 2.22 | -11.18, -2.49 | 0.002 | 0.004 |
| NSSI (number of days in the past 12 months; SITBI) | *χ^2^* (5) = 140.04, *p* < 0.001 | group | *χ^2^* (1) = 3.73, *p* = 0.053 |  |  |  |  |  |  |
|  |  | time | *χ^2^* (2) = 104.95, *p* < 0.001 | FU1 vs. baseline | -0.49 | 0.16 | -0.80, -0.19 | 0.002 | 0.004 |
|  |  |  |  | FU2 vs. FU1 | -1.25 | 0.17 | -1.58, -0.92 | < 0.001 | < 0.001 |
|  |  |  |  | FU2 vs. baseline | -1.74 | 0.17 | -2.08, -1.40 | < 0.001 | < 0.001 |
|  |  | (group)#(time) | *χ^2^* (2) = 10.63, *p* = 0.005 | (FU1 vs. baseline)#( inpatient/outpatient vs. outpatient) | 0.77 | 0.31 | 0.17, 1.38 | 0.012 | 0.019 |
|  |  |  |  | (FU2 vs. FU1)#( inpatient/outpatient vs. outpatient) | 0.24 | 0.33 | -0.40, 0.89 | 0.461 | 0.487 |
|  |  |  |  | (FU2 vs. baseline)#( inpatient/outpatient vs. outpatient) | 1.02 | 0.34 | 0.36, 1.68 | 0.002 | 0.005 |
|  |  |  | group@time | (inpatient/outpatient vs. outpatient)@baseline | -0.18 | 0.28 | -0.73,  0.36 | 0.514 | 0.533 |
|  |  |  |  | (inpatient/outpatient vs. outpatient)@FU1 | 0.59 | 0.28 | 0.04, 1.14 | 0.034 | 0.049 |
|  |  |  |  | (inpatient/outpatient vs. outpatient)@FU2 | 0.84 | 0.30 | 0.25, 1.42 | 0.005 | 0.009 |
| Suicidal thoughts (number of days in the past 12 months; SITBI) | *χ^2^* (5) = 30.96, *p* < 0.001 | group | *χ^2^* (1) = 8.32, *p* = 0.004 | inpatient/outpatient vs. outpatient | 0.41 | 0.14 | 0.13, 0.69 | 0.004 | 0.007 |
|  |  | time | *χ^2^* (2) = 17.04, *p* < 0.001 | FU1 vs. baseline | -0.14 | 0.17 | -0.48, 0.20 | 0.406 | 0.437 |
|  |  |  |  | FU2 vs. FU1 | -0.54 | 0.18 | -0.89, -0.20 | 0.002 | 0.004 |
|  |  |  |  | FU2 vs. baseline | -0.69 | 0.18 | -1.03, -0.34 | < 0.001 | < 0.001 |
|  |  | (group)#(time) | *χ^2^* (2) = 1.36, *p* = 0.506 |  |  |  |  |  |  |
| Suicide attempts (number in the past 12 months; SITBI) | *χ^2^* (5) = 41.43, *p* < 0.001 | group | *χ^2^* (1) = 1.21, *p* = 0.271 |  |  |  |  |  |  |
|  |  | time | *χ^2^* (2) = 33.16, *p* < 0.001 | FU1 vs. baseline | -0.48 | 0.21 | -0.89, -0.06 | 0.024 | 0.035 |
|  |  |  |  | FU2 vs. FU1 | -1.01 | 0.26 | -1.51, -0.51 | < 0.001 | < 0.001 |
|  |  |  |  | FU2 vs. baseline | -1.49 | 0.26 | -2.00, -0.98 | < 0.001 | < 0.001 |
|  |  | (group)#(time) | *χ^2^* (2) = 4.66, *p* = 0.097 |  |  |  |  |  |  |
| DIKJ | *χ^2^* (5) = 131.82, *p* < 0.001 | group | *χ^2^* (1) = 3.89, *p* = 0.049 | inpatient/outpatient vs. outpatient | 2.75 | 1.39 | 0.02, 5.48 | 0.049 | 0.067 |
|  |  | time | *χ^2^* (2) = 96.23, *p* < 0.001 | FU1 vs. baseline | -6.15 | 0.90 | -7.91, -4.39 | < 0.001 | < 0.001 |
|  |  |  |  | FU2 vs. FU1 | -3.14 | 1.00 | -5.09, -1.18 | 0.002 | 0.004 |
|  |  |  |  | FU2 vs. baseline | -9.29 | 0.99 | -11.24, -7.34 | < 0.001 | < 0.001 |
|  |  | (group)#(time) | *χ^2^* (2) = 6.63, *p* = 0.036 | (FU1 vs. baseline)#( inpatient/outpatient vs. outpatient) | 4.62 | 1.80 | 1.10, 8.13 | 0.010 | 0.017 |
|  |  |  |  | (FU2 vs. FU1)#( inpatient/outpatient vs. outpatient) | -2.62 | 2.00 | -6.53, 1.28 | 0.188 | 0.235 |
|  |  |  |  | (FU2 vs. baseline)#( inpatient/outpatient vs. outpatient) | 1.99 | 1.99 | -1.91, 5.89 | 0.317 | 0.348 |
|  |  |  | group@time | (inpatient/outpatient vs. outpatient)@baseline | 0.54 | 1.66 | -2.71, 3.79 | 0.743 | 0.756 |
|  |  |  |  | (inpatient/outpatient vs. outpatient)@FU1 | 5.16 | 1.74 | 1.74, 8.58 | 0.003 | 0.006 |
|  |  |  |  | (inpatient/outpatient vs. outpatient)@FU2 | 2.53 | 1.94 | -1.26, 6.33 | 0.190 | 0.228 |
| SCL-GSI | *χ^2^* (5) = 113.13, *p* < 0.001 | group | *χ^2^* (1) = 0.66, *p* = 0.417 |  |  |  |  |  |  |
|  |  | time | *χ^2^* (2) = 90.87, *p* < 0.001 | FU1 vs. baseline | -0.42 | 0.06 | -0.53, -0.30 | < 0.001 | < 0.001 |
|  |  |  |  | FU2 vs. FU1 | -0.10 | 0.06 | -0.21, 0.01 | 0.088 | 0.118 |
|  |  |  |  | FU2 vs. baseline | -0.52 | 0.06 | -0.63, -0.40 | < 0.001 | < 0.001 |
|  |  | (group)#(time) | *χ^2^* (2) = 4.19, *p* = 0.123 |  |  |  |  |  |  |
| KIDSCREEN-10 | *χ^2^* (5) = 83.20, *p* < 0.001 | group | *χ^2^* (1) = 3.49, *p* = 0.062 |  |  |  |  |  |  |
|  |  | time | *χ^2^* (2) = 59.53, *p* < 0.001 | FU1 vs. baseline | 3.59 | 0.73 | 2.16, 5.02 | < 0.001 | < 0.001 |
|  |  |  |  | FU2 vs. FU1 | 2.47 | 0.81 | 0.88, 4.06 | 0.002 | 0.005 |
|  |  |  |  | FU2 vs. baseline | 6.06 | 0.81 | 4.48, 7.64 | < 0.001 | < 0.001 |
|  |  | (group)#(time) | *χ^2^* (2) = 2.63, *p* = 0.269 |  |  |  |  |  |  |
| CGI-S | *χ^2^* (5) = 294.81, *p* < 0.001 | group | *χ^2^* (1) = 14.06, *p* < 0.001 | inpatient/outpatient vs. outpatient | 0.56 | 0.15 | 0.27, 0.86 | < 0.001 | < 0.001 |
|  |  | time | *χ^2^* (2) = 248.56, *p* < 0.001 | FU1 vs. baseline | -1.38 | 0.11 | -1.60, -1.16 | < 0.001 | < 0.001 |
|  |  |  |  | FU2 vs. FU1 | -0.27 | 0.11 | -0.49, -0.06 | 0.013 | 0.020 |
|  |  |  |  | FU2 vs. baseline | -1.65 | 0.11 | -1.87, -1.43 | < 0.001 | < 0.001 |
|  |  | (group)#(time) | *χ^2^* (2) = 3.07, *p* = 0.215 |  |  |  |  |  |  |

*Notes*. P-values adjusted for multiple comparison according to Benjamini-Hochberg. BPD, borderline personality disorder; CGI-S: Clinical Global Impression Scale-Severity; DIKJ, Depression Inventory for Children and Adolescents; FU1: follow-up 1, 12 months after baseline; FU2: follow-up 2, 24 months after baseline; GAF, Global Assessment of Functioning; NSSI: non-suicidal self-injury; SCLGSI, Symptom-Checklist-90 Revised; SITBI, Self-Injurious Thoughts and Behaviors Interview.

***Table 3****.* Sensitivity analysis B (Generalized and mixed models based on raw data of only those participants who had no missing values with inverted probability weights to adjust for initial differences in patient characteristics)

| **Outcome** | **Model fit** | **Main effects** | | **Contrasts** | | | | | |
| --- | --- | --- | --- | --- | --- | --- | --- | --- | --- |
|  |  | Predictors |  | Predictors | β | SE | 95% CI | p value | p value adjusted for multiple comparisons |
| BPD (number of criteria) | *χ^2^* (5) = 44.31, *p* < 0.001 | group | *χ^2^* (1) = 0.49, *p* = 0.482 |  |  |  |  |  |  |
|  |  | time | *χ^2^* (2) = 21.75, *p* < 0.001 | FU1 vs. baseline | -0.26 | 0.11 | -0.47, -0.05 | 0.013 | 0.025 |
|  |  |  |  | FU2 vs. FU1 | -0.40 | 0.11 | -0.62, -0.19 | < 0.001 | 0.001 |
|  |  |  |  | FU2 vs. baseline | -0.67 | 0.14 | -0.95, -0.38 | < 0.001 | < 0.001 |
|  |  | (group)#(time) | *χ^2^* (2) = 2.76, *p* = 0.251 |  |  |  |  |  |  |
| GAF | *χ^2^* (5) = 166.19, *p* < 0.001 | group | *χ^2^* (1) = 5.59, *p* = 0.018 | inpatient/outpatient vs. outpatient | -4.90 | 2.07 | -8.97, -0.84 | 0.018 | 0.032 |
|  |  | time | *χ^2^* (2) = 92.99, *p* < 0.001 | FU1 vs. baseline | 11.06 | 1.40 | 8.31, 13.80 | < 0.001 | < 0.001 |
|  |  |  |  | FU2 vs. FU1 | 6.06 | 1.67 | 2.79, 9.32 | < 0.001 | 0.001 |
|  |  |  |  | FU2 vs. baseline | 17.11 | 1.92 | 13.34, 20.88 | < 0.001 | < 0.001 |
|  |  | (group)#(time) | *χ^2^* (2) = 14.86, *p* = 0.001 | (FU1 vs. baseline)#( inpatient/outpatient vs. outpatient) | -10.59 | 2.80 | -16.08, -5.10 | < 0.001 | < 0.001 |
|  |  |  |  | (FU2 vs. FU1)#( inpatient/outpatient vs. outpatient) | 5.22 | 3.33 | -1.31, 11.76 | 0.117 | 0.169 |
|  |  |  |  | (FU2 vs. baseline)#( inpatient/outpatient vs. outpatient) | -5.37 | 3.85 | -12.01, 2.17 | 0.163 | 0.229 |
|  |  |  | group@time | (inpatient/outpatient vs. outpatient)@baseline | 0.42 | 2.03 | -3.57, 4.41 | 0.837 | 0.853 |
|  |  |  |  | (inpatient/outpatient vs. outpatient)@FU1 | -10.17 | 2.40 | -14.88, -5.47 | < 0.001 | < 0.001 |
|  |  |  |  | (inpatient/outpatient vs. outpatient)@FU2 | -4.95 | 3.77 | -12.34, 2.45 | 0.190 | 0.253 |
| NSSI (number of days in the past 12 months; SITBI) | *χ^2^* (5) = 118.10, *p* < 0.001 | group | *χ^2^* (1) = 0.45, *p* = 0.503 |  |  |  |  |  |  |
|  |  | time | *χ^2^* (2) = 111.58, *p* < 0.001 | FU1 vs. baseline | -0.69 | 0.16 | -1.01, -0.37 | < 0.001 | < 0.001 |
|  |  |  |  | FU2 vs. FU1 | -1.56 | 0.22 | -1.98, -1.13 | < 0.001 | < 0.001 |
|  |  |  |  | FU2 vs. baseline | -2.25 | 0.21 | -2.67, -1.83 | < 0.001 | < 0.001 |
|  |  | (group)#(time) | *χ^2^* (2) = 5.17, *p* = 0.076 | (FU1 vs. baseline)#( inpatient/outpatient vs. outpatient) | 0.59 | 0.33 | -0.04, 1.23 | 0.068 | 0.100 |
|  |  |  |  | (FU2 vs. FU1)#( inpatient/outpatient vs. outpatient) | 0.21 | 0.44 | -0.65, 1.06 | 0.636 | 0.675 |
|  |  |  |  | (FU2 vs. baseline)#( inpatient/outpatient vs. outpatient) | 0.80 | 0.42 | -0.03, 1.63 | 0.058 | 0.089 |
|  |  |  | group@time | (inpatient/outpatient vs. outpatient)@baseline | -0.23 | 0.35 | -0.92, 0.46 | 0.519 | 0.586 |
|  |  |  |  | (inpatient/outpatient vs. outpatient)@FU1 | 0.37 | 0.42 | -0.45, 1.18 | 0.377 | 0.445 |
|  |  |  |  | (inpatient/outpatient vs. outpatient)@FU2 | 0.57 | 0.49 | -0.39, 1.54 | 0.242 | 0.300 |
| Suicidal thoughts (number of days in the past 12 months; SITBI) | *χ^2^* (5) = 49.35, *p* < 0.001 | group | *χ^2^* (1) = 0.89, *p* = 0.345 |  |  |  |  |  |  |
|  |  | time | *χ^2^* (2) = 19.81, *p* < 0.001 | FU1 vs. baseline | -0.31 | 0.16 | -0.62, 0.01 | 0.056 | 0.088 |
|  |  |  |  | FU2 vs. FU1 | -0.85 | 0.21 | -1.27, -0.43 | < 0.001 | < 0.001 |
|  |  |  |  | FU2 vs. baseline | -1.16 | 0.27 | -1.68, -0.64 | < 0.001 | < 0.001 |
|  |  | (group)#(time) | *χ^2^* (2) = 3.57, *p* = 0.167 |  |  |  |  |  |  |
| Suicide attempts (number in the past 12 months; SITBI) | *χ^2^* (5) = 38.65, *p* < 0.001 | group | *χ^2^* (1) = 2.54, *p* = 0.111 |  |  |  |  |  |  |
|  |  | time | *χ^2^* (2) = 13.68, *p* = 0.001 | FU1 vs. baseline | -0.55 | 0.25 | -1.05, -0.06 | 0.029 | 0.049 |
|  |  |  |  | FU2 vs. FU1 | -0.94 | 0.33 | -1.58, -0.29 | 0.005 | 0.010 |
|  |  |  |  | FU2 vs. baseline | -1.49 | 0.40 | -2.28, -0.70 | < 0.001 | 0.001 |
|  |  | (group)#(time) | *χ^2^* (2) = 6.73, *p* = 0.035 | (FU1 vs. baseline)#( inpatient/outpatient vs. outpatient) | 1.21 | 0.53 | 0.17, 2.24 | 0.022 | 0.038 |
|  |  |  |  | (FU2 vs. FU1)#( inpatient/outpatient vs. outpatient) | -0.79 | 0.66 | -2.09, 0.51 | 0.232 | 0.294 |
|  |  |  |  | (FU2 vs. baseline)#( inpatient/outpatient vs. outpatient) | 0.41 | 0.85 | -1.25, 2.08 | 0.627 | 0.679 |
|  |  |  | group@time | (inpatient/outpatient vs. outpatient)@baseline | 0.19 | 0.31 | -0.42, 0.80 | 0.535 | 0.592 |
|  |  |  |  | (inpatient/outpatient vs. outpatient)@FU1 | 1.40 | 0.52 | 0.38, 2.42 | 0.007 | 0.014 |
|  |  |  |  | (inpatient/outpatient vs. outpatient)@FU2 | 0.61 | 0.87 | -1.09, 2.30 | 0.484 | 0.559 |
| DIKJ | *χ^2^* (5) = 74.36, *p* < 0.001 | group | *χ^2^* (1) = 0.94, *p* = 0.332 |  |  |  |  |  |  |
|  |  | time | *χ^2^* (2) = 33.23, *p* < 0.001 | FU1 vs. baseline | -6.82 | 1.35 | -9.47, -4.18 | < 0.001 | < 0.001 |
|  |  |  |  | FU2 vs. FU1 | -2.77 | 1.15 | -5.03, -0.50 | 0.017 | 0.031 |
|  |  |  |  | FU2 vs. baseline | -9.59 | 1.71 | -12.94, -6.23 | < 0.001 | < 0.001 |
|  |  | (group)#(time) | *χ^2^* (2) = 1.83, *p* = 0.400 |  |  |  |  |  |  |
| SCL-GSI | *χ^2^* (5) = 74.18, *p* < 0.001 | group | *χ^2^* (1) = 0.43, *p* = 0.512 |  |  |  |  |  |  |
|  |  | time | *χ^2^* (2) = 38.80, *p* < 0.001 | FU1 vs. baseline | -0.50 | 0.08 | -0.66, -0.33 | < 0.001 | < 0.001 |
|  |  |  |  | FU2 vs. FU1 | -0.03 | 0.07 | -0.16, 0.10 | 0.657 | 0.683 |
|  |  |  |  | FU2 vs. baseline | -0.52 | 0.09 | -0.71, -0.34 | < 0.001 | < 0.001 |
|  |  | (group)#(time) | *χ^2^* (2) = 0.65, *p* = 0.724 |  |  |  |  |  |  |
| KIDSCREEN-10 | *χ^2^* (5) = 29.43, *p* < 0.001 | group | *χ^2^* (1) = 1.16, *p* = 0.281 |  |  |  |  |  |  |
|  |  | time | *χ^2^* (2) = 17.22, *p* < 0.001 | FU1 vs. baseline | 2.97 | 0.88 | 1.25, 4.69 | < 0.001 | 0.002 |
|  |  |  |  | FU2 vs. FU1 | 1.88 | 0.89 | 0.14, 3.62 | 0.034 | 0.055 |
|  |  |  |  | FU2 vs. baseline | 4.85 | 1.20 | 2.51, 7.19 | < 0.001 | < 0.001 |
|  |  | (group)#(time) | *χ^2^* (2) = 1.54, *p* = 0.463 |  |  |  |  |  |  |
| CGI-S | *χ^2^* (5) = 281.18, *p* < 0.001 | group | *χ^2^* (1) = 2.96, *p* = 0.086 |  |  |  |  |  |  |
|  |  | time | *χ^2^* (2) = 182.84, *p* < 0.001 | FU1 vs. baseline | -1.38 | 0.11 | -1.60, -1.16 | < 0.001 | < 0.001 |
|  |  |  |  | FU2 vs. FU1 | -0.37 | 0.13 | -0.62, -0.12 | 0.004 | 0.009 |
|  |  |  |  | FU2 vs. baseline | -1.75 | 0.15 | -2.04, -1.45 | < 0.001 | < 0.001 |
|  |  | (group)#(time) | *χ^2^* (2) = 9.94, *p* = 0.007 | (FU1 vs. baseline)#( inpatient/outpatient vs. outpatient) | 0.70 | 0.23 | 0.25, 1.14 | 0.002 | 0.005 |
|  |  |  |  | (FU2 vs. FU1)#( inpatient/outpatient vs. outpatient) | -0.34 | 0.25 | -0.84, 0.16 | 0.188 | 0.257 |
|  |  |  |  | (FU2 vs. baseline)#( inpatient/outpatient vs. outpatient) | 0.36 | 0.30 | -0.23, 0.95 | 0.231 | 0.301 |
|  |  |  | group@time | (inpatient/outpatient vs. outpatient)@baseline | 0.00 | 0.16 | -0.31, 0.31 | 0.993 | 0.993 |
|  |  |  |  | (inpatient/outpatient vs. outpatient)@FU1 | 0.69 | 0.25 | 0.20, 1.19 | 0.006 | 0.013 |
|  |  |  |  | (inpatient/outpatient vs. outpatient)@FU2 | 0.36 | 0.32 | -0.27, 0.99 | 0.267 | 0.323 |

*Notes*. P-values adjusted for multiple comparison according to Benjamini-Hochberg. BPD, borderline personality disorder; CGI-S: Clinical Global Impression Scale-Severity; DIKJ, Depression Inventory for Children and Adolescents; FU1: follow-up 1, 12 months after baseline; FU2: follow-up 2, 24 months after baseline; GAF, Global Assessment of Functioning; NSSI: non-suicidal self-injury; SCLGSI, Symptom-Checklist-90 Revised; SITBI, Self-Injurious Thoughts and Behaviors Interview.
